# Genome Sequences of Subcluster M2 Mycobacteriophages Estes and Aziz

**DOI:** 10.1128/MRA.00104-21

**Published:** 2021-03-11

**Authors:** Sara K. Fitzgerald, Eleanor H. Johnson, Sophie H. R. Storz, Claire Ballard, Samantha Battaglia, Mikelle Boice, Joshua Bramwell-Butcher, Megan Dedinsky, Joshua DeKlotz, Izabel Diaz, Andrew Engley, Lindsey Ernst, Erica Gonzales, Alyssa Groscost, Paige Grosser, Alicia Haider, Morgan Harrison, Kurt Husler, Jalisa Lau, Melina Monlux, Jason Paratore, Trevor Ruesch, Mikaela Schlesinger, Anthony Scholes, Marianne K. Poxleitner, Kirk R. Anders

**Affiliations:** a Department of Biology, Gonzaga University, Spokane, Washington, USA; Portland State University

## Abstract

Estes and Aziz are mycobacteriophages that were isolated on *Mycolicibacterium smegmatis* mc^2^155 at room temperature from soil samples collected in Spokane, WA. Their genome sequences are 83,601 and 83,412 bp long, respectively, and they are members of subcluster M2. Each contains 21 tRNA genes and short conserved repeats characteristic of cluster M phages.

## ANNOUNCEMENT

Bacteriophages represent a vast degree of genetic diversity, the majority of which remains to be discovered (1). The study of this genetic variation provides valuable insight into viral genome structure, organization, and gene function and may contribute to phage therapy and other applications. To contribute to this effort, we present here the genome sequences of bacteriophages Estes and Aziz, isolated as part of the SEA-PHAGES program at Gonzaga University (2, 3).

Estes and Aziz were isolated as turbid plaques on *Mycolicibacterium smegmatis* mc^2^155 from two soil samples taken from distinct locations on the Gonzaga campus in Spokane, WA. The soil was enriched by incubating with M. smegmatis for 7 days in growth medium, which was then filtered (0.22 μm) and plated onto lawns at room temperature (22°C). Lysates were collected from plates of purified plaques. DNA was purified from lysates with the Promega Wizard DNA cleanup system. Sequencing libraries were prepared using the NEB Ultra II FS library prep kit with dual-indexed barcoding and then pooled and sequenced using an Illumina MiSeq instrument to produce single-end, 150-bp reads. Newbler v2.9 (4) and Consed v29 (5) were used to assemble and finish the genome sequences as described (6). The genomic termini were determined using PAUSE (https://cpt.tamu.edu/computer-resources/pause/). The average depths of coverage were 808× for Estes and 749× for Aziz. The Estes and Aziz genome sequences are 83,601 and 83,412 bp long, with GC contents of 60.9% and 60.7%, respectively. Both contain 3′ overhanging ends with the sequence ACCCCATGCAA. Estes and Aziz share 92.5% average nucleotide identity (ANI), determined by DNA Master v5.23.6 (http://cobamide2.bio.pitt.edu). Both are most closely related to subcluster M2 phages (https://phagesdb.org/subclusters/M2). Estes shares 94.7% ANI with MrMagoo, and Aziz shares 99.8% ANI with GenevaB15.

**FIG 1:**
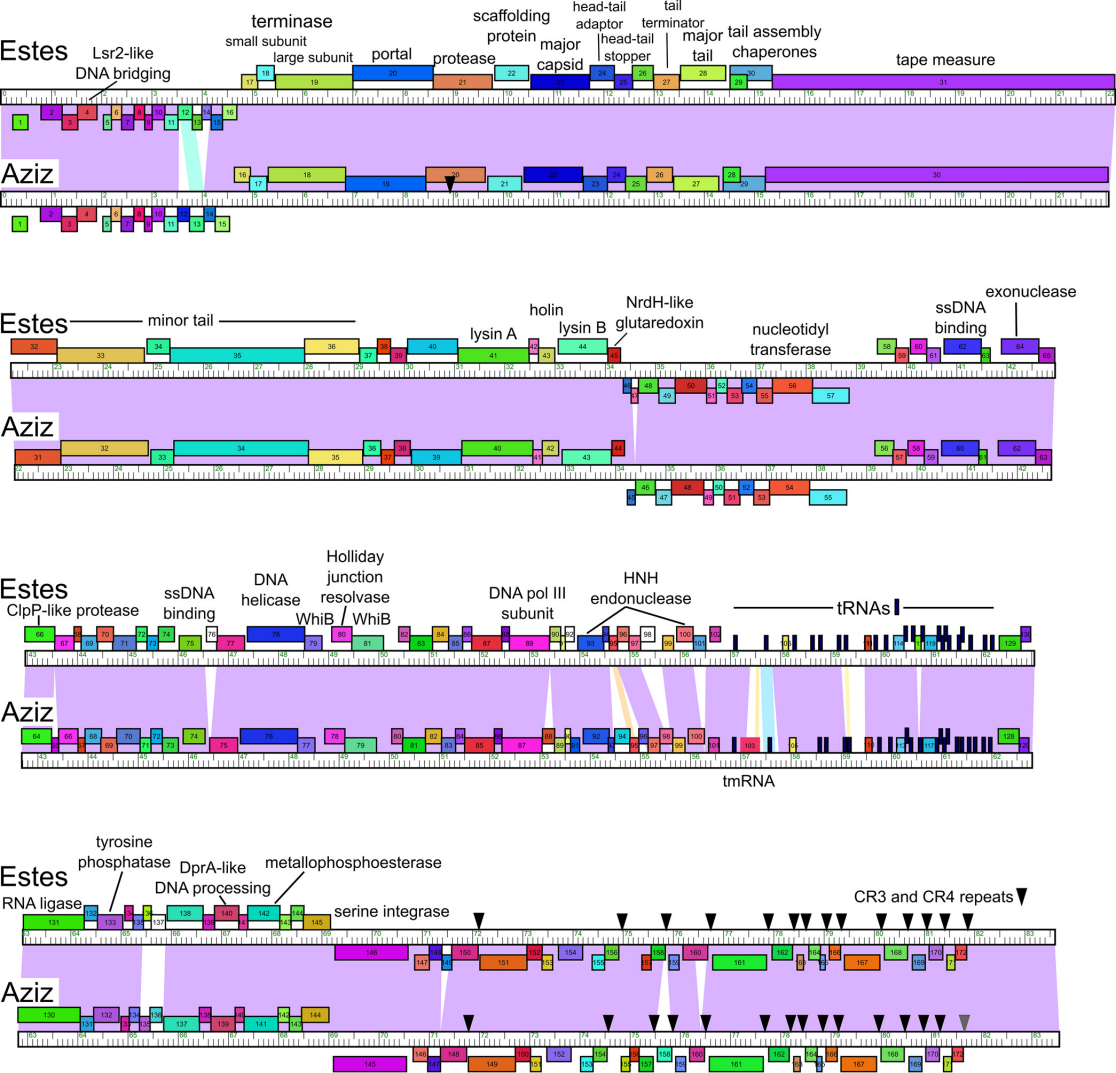
Genome maps of Estes and Aziz. The genome sequences are shown aligned with each other and drawn in four tiers. The ruler for each genome shows the length in kilobases. Genes are shown as boxes above and below each genome, indicating rightward- and leftward-transcribed genes, respectively. Genes are colored according to phamily designation, determined by Phamerator and the Actino_Draft database v391 (14, 16). The white boxes indicate genes with no close relatives, and the narrow black boxes indicate tRNA genes. Functions are predicted for genes in Estes and also apply to the identically colored orthologs in Aziz. Shading between the genomes displays nucleotide sequence similarity determined by BLASTN, with the most similar shown in violet and the least similar in yellow. Sequences conforming to CR3 and/or CR4 repeats (15) are shown as black arrowheads. The gray arrowhead indicates a match to CR3 with two base pair mismatches.

The genome sequences were manually annotated using Glimmer v3.02 (7), GeneMark v2.5p (8), DNA Master, Starterator v1.2 (https://github.com/SEA-PHAGES/starterator), ARAGORN v1.2 (9), and tRNAscan-SE v2.0 (10) to predict protein- and RNA-coding genes. Gene functions were predicted using HHPred v3.2 (11), TmHMM v2.0 (12), NCBI BLAST v2.10 (13), and the Actinobacteriophage Database (14). Default parameters were used for all software unless otherwise specified. Estes and Aziz contain 151 and 150 protein-coding genes, respectively, 40 of which were assigned functions. Both genomes display the following noncanonical genome architectures and features consistent with those of cluster M phages: a block of leftward-transcribed genes to the left of the terminase and structural genes, a 5-kb region containing 21 tRNA genes (and a transfer-messenger RNA [tmRNA] gene in Aziz), an integration cassette located 13 kb from the genome’s right end, and conserved repeats between many genes in the rightmost region (Fig. 1) (15).

Estes and Aziz share 20 tRNA genes, 19 of which are conserved across subcluster M2 with identical anticodons. In addition, Estes shares a tRNA^Leu^ gene solely with GenevaB15 (GenBank accession number MF319184), while Aziz shares a tRNA^Ala^ gene with GenevaB15, GardenSalsa (KY783914), and MrMagoo (KY223999). Estes and Aziz contain the conserved repeats CR1 through CR4 in similar numbers and locations as described in cluster M phages, including stem-loop sequences upstream of CR3 and CR4 (15). Relative to the genome sequences in reference 15, Aziz gene *158* and Estes gene *172* are insertions with associated copies of CR3. Orthologs of both genes are present in GenevaB15, and orthologs of Estes gene *172* can additionally be found in MrMagoo and Aziz. The inserted CR3 in Aziz, however, contains two mismatches relative to the consensus.

### Data availability.

The GenBank and Sequence Read Archive accession numbers are MT657341 and SRR13020420 for Estes and MT658802 and SRR13020421 for Aziz, respectively.
